# Mastectomy Skin Flap Perfusion Assessment Prior to Breast Reconstruction: A Narrative Review

**DOI:** 10.3390/jpm14090946

**Published:** 2024-09-06

**Authors:** Alex Victor Orădan, Alexandru Valentin Georgescu, Alexandru Ilie-Ene, Alma Andreea Corpodean, Teodora Paula Juncan, Maximilian Vlad Muntean

**Affiliations:** 1 Department of Surgery—Plastic and Reconstructive Surgery, “Iuliu Hațieganu” University of Medicine and Pharmacy, 400012 Cluj-Napoca, Romania; oradan.alex@umfcluj.ro (A.V.O.);; 2 Department of Plastic and Reconstructive Surgery, Clinical Rehabilitation Hospital, 400066 Cluj-Napoca, Romania; 3 Department of Plastic and Reconstructive Surgery, “Prof. Dr. I. Chiricuță” Institute of Oncology, 400015 Cluj-Napoca, Romania; 4 Department of Surgery, First Surgical Clinic, Emergency County Hospital, 400006 Cluj-Napoca, Romania; 5 Department of Surgery, “Iuliu Hațieganu” University of Medicine and Pharmacy, 400012 Cluj-Napoca, Romania; 6 Department of Plastic and Reconstructive Surgery, First Surgical Clinic, Emergency County Hospital, 400006 Cluj-Napoca, Romania

**Keywords:** mastectomy flap, perfusion assessment, breast reconstruction, indocyanine green

## Abstract

Background: Predicting the viability of the skin flaps after mastectomy is of high importance and significance in immediate breast reconstruction. Numerous methods have been used and are readily available. This review aims to describe and compare the current preferred perfusion assessment tools. Methods: Four major scientific databases—Web of Science, PubMed, Embase, and Scopus—were consulted to retrieve reviews, meta-analyses, clinical trials, experimental studies, and case reports focused on skin flap perfusion assessment following mastectomy. English-language articles published within the last 10 years were included. The most recent search was conducted on 31 July 2024. Results: A summary focused on the relevant information of all included studies was drafted, and the results of the studies have been synthetized and compared. A total of 58 studies have been included in this review. Conclusion: Indocyanine green angiography (ICG-A) is the preferred and most-used method of evaluating perfusion, especially in high-risk patients, while new technologies show promising results and might be of great interest in the future. Perfusion assessment tools complement and should not replace clinical evaluation.

## 1. Introduction

Breast cancer continues to be the most common type of cancer in women worldwide [1]. Since the introduction of skin-sparing mastectomy and immediate reconstruction techniques, rates have increased and can significantly improve the quality of life for the patient [2,3,4]. By preserving the patient’s skin envelope and inframammary fold, an easier one-stage reconstruction can be obtained with superior aesthetic results [5]. This leads to higher levels of patient satisfaction, psychological outcomes, and cost-effectiveness [6].

Mastectomy skin flap necrosis (MSFN) is the most common complication in skin-sparing techniques, with reported rates as high as 30% [7]. Ischemic complication can lead to skin breakdown with implant exposure, explantation, infection, an overall delay in healing and adjuvant therapy, or the complete failure of the reconstruction, all together with a reduced quality of life [5,8]. Risk factors such as age, smoking, diabetes mellitus, high body mass index, high mastectomy specimen weight, and previous radiotherapy can also affect the outcomes of reconstruction [5]. Translating the intraoperative measurements of ischemia has proven challenging and is continuing to be a subject of interest; thus, a precise and reliable method of evaluating the mastectomy skin flap perfusion is paramount [8].

Initially, tissue perfusion was assessed intraoperatively via clinical evaluation only, observing skin color, capillary refill time, and bleeding [5]. Relying strictly on clinical assessment is prone to over-resection and may limit the reconstructive options. Fluorescence angiography with indocyanine green (ICG-A) or fluorescein has been used before in multiple surgical specialties besides plastic surgery [9]. The process relies on the injection of a dye intravenously, which emits infrared light when excited by a light source. This allows for an accurate real-time evaluation of tissue perfusion and can predict tissue viability. High cost, device bulkiness, and risk of allergic reactions are among the disadvantages of these methods [10,11,12]. Near-infrared-spectroscopy-based systems utilize near-infrared light to assess tissue oxygenation saturation. T.Ox and Intra.Ox (ViOptix Inc., Newark, CA, USA), initially used for free flap monitoring, measure tissue oxygenation in one point. SnapshotNIR (Kent Imaging, Calgary, AB, Canada) can provide patterns of tissue oxygenation within an area and produce a map of perfusion [12]. Similarly, hyperspectral imaging (HSI), a form of spectroscopy, uses light to measure parameters like oxygenation, water content, and hemoglobin content of the tissue and produce a map of tissue perfusion [13,14]. Thermal imaging by means of a handheld device (FLIR ONE, FLIR Systems, Wilsonville, OR, USA) measures skin surface temperature, which can also be used as a perfusion indicator in mastectomy flaps [15]. The different methods of perfusion assessment are summarized in Table 1.

The purpose of this narrative review is to describe and compare the current methods used for evaluating mastectomy skin flap perfusion after mastectomy and before reconstruction, each with its own advantages and disadvantages.

## 2. Materials and Methods

This review addresses a clearly focused issue, adopting the population, intervention, comparison, and outcome (PICO) method, and is conducted in accordance with the statement of the preferred reporting of systematic reviews and meta-analysis checklist (PRISMA 2020).

### 2.1. Eligibility Criteria

Population: We selected articles with human or animal subjects. The following types of study were analyzed: reviews and meta-analyses, clinical trials, case reports, and experimental descriptions.

Intervention: We considered all studies that reported on mastectomy skin flap perfusion assessment. We excluded those where skin perfusion was assessed in any other type of surgical flaps, and studies where no skin flap perfusion assessment was made.

Comparison: The following mastectomy skin flap perfusion assessment methods were compared: (1) clinical examination; (2) angiography (indocyanine green, fluorescein); (3) spectroscopy (near infrared oximetry, HSI); and (4) thermal imaging.

Outcomes: Advantages and disadvantages of the compared mastectomy skin flap perfusion methods; complications and patient-specific reported outcomes and aesthetic results. 

Only English-written studies published in the past 10 years were considered eligible.

### 2.2. Search Strategy

Web of Science, PubMed, Embase, and Scopus databases were used. The last inquiry was conducted on 31 July 2024.

The following keywords were included in the search strategy: “mastectomy flap”’; “perfusion”; “assessment”. To improve the accuracy of the results yielded, the search was aimed at the title and the abstract. A filter for language was applied, selecting all English-written articles. The following search lines were generated and used:

- Web of Science: ((TS = (mastectomy flap)) AND TS = (perfusion)) AND TS = (assessment).
- PubMed: ((assessment [Title/Abstract]) AND (perfusion [Title/Abstract])) AND (mastectomy flap [Title/Abstract]).
- Embase: (“mastectomy”/exp OR “mastectomy”) AND (“flap”/exp OR “flap”) AND (“perfusion”/exp OR “perfusion”) AND (“assessment”/exp OR “assessment”).
- Scopus: TITLE-ABS-KEY (mastectomy AND flap W/255 perfusion W/255 assessment).

### 2.3. Study Selection and Data Collection Process

**Figure 1:**
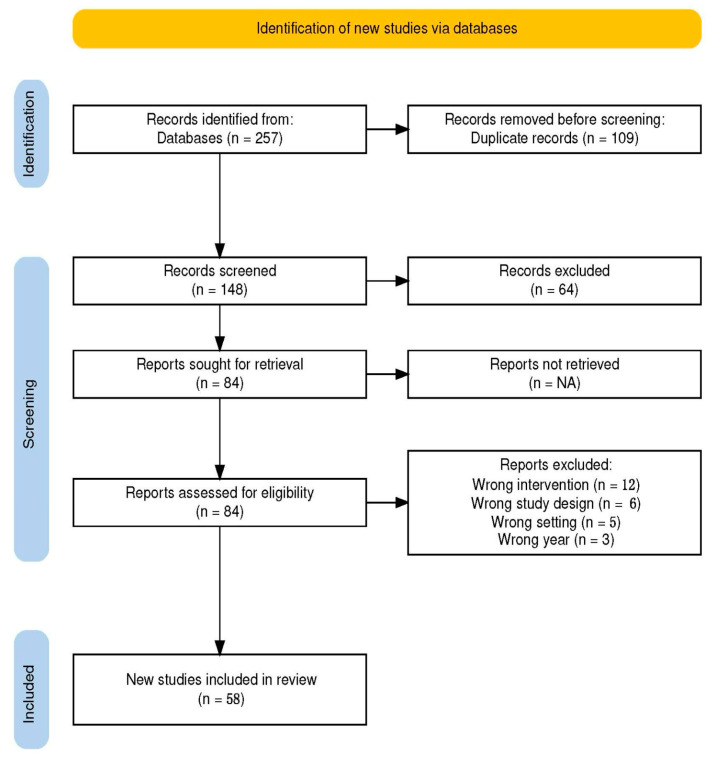
PRISMA flow-diagram.

A cumulative number of 257 scientific articles was yielded by the refined search. After duplicates (n = 108) removal, the 149 articles were independently screened by two researchers (AO and AIE). In the first phase, the articles were screened via title and abstract review. The conflicts between the two researchers were resolved by consulting a third researcher (AC). The resulting 84 articles were full-text screened by the same two researchers. Disagreements were resolved in the same fashion. A total of 58 eligible articles were considered for analysis. A web-based collaboration software platform that streamlines the production of systematic reviews was used for this task [16]. Figure 1 is a PRISMA flow-diagram that displays the study selection process.

Data extraction from each study that was conducted by AO. The resulting information was validated by a second researcher (MM) prior to it being included in the analysis.

The sought outcome was the identification of the optimal mastectomy skin flap perfusion assessment tool. This was considered a critical outcome domain. We anticipated a variety of skin flap perfusion assessment methods. The means of using each method was also noted.

We collected data on the following:

- The report: authors; date of publication.
- The research design and features: article type; period from which the data were collected in the included studies.
- The materials: type of system used for skin flap perfusion assessment.
- The intervention: skin flap perfusion assessment technique.
- The results: clinical and functional outcome of skin flap technique used; advantages and disadvantages of each technique.

## 3. Results

The 58 articles included in the review were the following: 19 reviews, 32 clinical trials, 4 experimental, and 3 case reports. In the following paragraphs, a summary of the main outcomes of the studies are presented.

### 3.1. Reviews

A thorough description of multiple methods with which to evaluate the intraoperative perfusion of mastectomy skin flaps was conducted by Khavanin et al., each with their own advantages and disadvantages [8]. FA and ICG-A are considered key decision-making tools in reducing skin necrosis [17,18,19]. The majority of the reviews emphasize the utility of ICG-A and main advantages over clinical examination and FA [5,20,21,22,23]. The use of HSI in the assessment of mastectomy flap perfusion was also described in a few studies [13,24]. A detailed description of all the review papers is provided in Table 2.

### 3.2. Clinical Trials

Most of the clinical trials focus on the use of ICG-A and point out a reduction in complication rate and costs [12,26,32]. ICG-A has proven its efficacy in direct-to-implant (DTI) reconstruction, especially in high-risk cases, as well as in oncoplastic breast surgery [33,34,35,36,37,38]. Moreover, compared to clinical judgment, it is considered superior; it offers more information about the perfusion but it should not replace it as it tends to overpredict necrosis [39,40,41,42]. ICG-A can also be used preoperatively to assess the vasculature of the breast envelope (Yang, Geletzke). FA also plays a role in perfusion assessment, although it is considered inferior [43,44]. Clinical assessment continues to play an important role in perfusion evaluation [45,46]. Although not many studies focus on other methods, thermal imaging and spectroscopy devices have proven their efficacy [12,14,15,42,47]. A detailed description of all the clinical trials is provided in Table 3.

### 3.3. Case Reports

ICG-A was used intraoperatively to assess perfusion, and conservative measures like skin warming and expander deflation were taken [57]. Marques et al. report two cases of NSM and immediate implant reconstruction in which ICG-A was used. ICG-A and clinical judgment provides a real-time assessment of tissue perfusion, although cut-off values for perfusion related to MSFN remains to be investigated [58]. In one case report, an individualized approach resulted in optimal outcomes for the patient with the use of ICG-A after NSM to prevent postoperative ischemic complications [59].

### 3.4. Experimental Studies

Warming the flap intraoperatively to achieve maximum vasodilation before performing ICG-A mimics the microcirculatory environment encountered at 24 h and better predicts flap survival [60]. Tissue oximetry and thermal imaging were also more capable of predicting skin necrosis compared to ICG-A and represented potentially less-expensive and more readily available alternatives for objective perfusion assessment, although they are less accurate [61]. Multispectral reflectance imaging’s predictive capability with respect to tissue necrosis can be greater than or equal to that of indocyanine green angiography [24]. In a preclinical study, near-infrared spectroscopy using the ViOptix Intra.Ox (ViOptix, Inc., Fremont, CA, USA) detected significant differences in tissue oxygenation saturation, which are associated with the risk for flap necrosis [62].

## 4. Discussion

The evaluation of skin perfusion after mastectomy dictates the success of breast reconstruction and is an important tool when striving for a complication-free outcome. The selection of appropriate reconstructive options is determined via a thorough intraoperative evaluation of flaps using clinical and additional methods to minimize complications and maximize both functional and cosmetic outcomes in breast reconstruction [63]. Several reviews and meta-analyses point out the utility of imaging modalities to augment the clinical assessment. Alongside patient specific factors and a dissection technique that allows for the preservation of the vasculature, clinical examination and the use of imaging and adjunctive technologies can lead to a better evaluation of the mastectomy flap perfusion prior to reconstruction in order to obtain better results [45,64].

Skin necrosis is considered an important short-term complication [17]. A good perfusion assessment technique should be able to distinguish between poorly perfused tissue that will survive and tissue that is destined for necrosis [5]. ICG-A and FA are considered key decision-making tools in reducing skin necrosis, although other methods have also been used successful [12,13,15,17,24,39,46,47,61,62,65]. Overall, a good perfusion assessment tool should finally guide the surgeon to change their strategy by decreasing expander volumes, resect poorly perfused tissue, convert from implant to expander, or even delay the reconstruction [17,57].

ICG-A is the most frequently used method which aids in perfusion assessments during plastic and reconstructive surgery and should no longer be considered experimental. It allows the surgeon to assess perfusion and achieve the best outcomes for the patient [28,30,48,59]. It is universally accepted that ICG-A is more reliable and more objective than clinical judgment alone but cannot and should not entirely replace it [5,22,40,54]. When concerns arise in clinical evaluation, ICG-A is useful as a supplement rather than a substitute for clinical assessment in flap perfusion evaluation [21,34,35,36,48]. Therefore, clinical examination coupled with ICG-A can enhance the accuracy of predicting mastectomy flap necrosis [20,23,25,31]. ICG-A may be most useful when used on patients at high risk of necrosis, such as those with heavy mastectomy breast weight, those with a high body mass index, and smokers [34]. In low-risk cases, it would result in a more aggressive resection compared to clinical examination and should only be used when clinical examination is marginal [41]. The use of ICG-A tends to over-predict skin necrosis and lead to unnecessary resection [5,41,60]. Since the introduction of ICG-A in mastectomy flap perfusion evaluation, there has been a significant drop in the complication rate, with reduced rates of mastectomy flap necrosis [11,22,26,27,32,51]. With fewer returns to the operating theater and no reoperation, the overall costs of such procedures have dropped [11,27,40]. In addition, more information about perfusion can lead to a more conservative approach to reconstruction and can guide the surgeon to perform a delayed reconstruction if the perfusion is scarce [39]. ICG-A can also improve outcomes such as increased expander fill volumes and shorter waiting time to final reconstruction [33]. In the recent transition from subpectoral to prepectoral implant placement, DTI reconstruction has become safer with ICG-A [50].

Moreover, ICG-A can also be used in oncoplastic breast surgery to assess perfusion and select perforators or before mastectomy to assess the dominant vasculature of the breast for optimal reconstruction and improve outcomes [37,38,42]. Tissue tension from inadequate implant volume may cause hypoperfusion and mastectomy flap ischemia. According to Yang et al., ICG-A was used to analyze the changes in tissue perfusion in conjunction with the tension level. It was objectively proven that tissue perfusion deteriorates as the tension applied to the flap increases [52]. Refinement of the used software can provide accurate values for perfusion, and a quantitative assessment of perfusion can more accurately guide the decision-making process intraoperatively [25,53]. Factors that influence and optimize ICG-A intensity are a weight-adjusted dose, the use of a recommended working distance, and imaging head positioning between 60 and 90 degrees [55]. A scoring system and cut-off values for perfusion assessment were also described to prevent complications [55,58]. Based on ICG-A, four types of ischemia patterns were described and used in the decision-making process to delay the reconstruction [34,43]. Furthermore, in the era of artificial intelligence, machine learning algorithms based on ICG-A videos can further guide the resection to optimize the outcomes [56]. Although the benefits of ICG-A had a significant impact on breast reconstruction, high-quality randomized controlled trials that compare the use of ICG-A to clinical evaluation are needed to further support its use [27,29].

Alongside ICG-A, FA has also been used to assess mastectomy flap perfusion and can be helpful in high-risk patients [18,19,44,49]. FA lacks sufficient objectivity and may guide the resection of intermediately perfused skin flaps. This may also be adequately assessed by clinical judgment [5]. Overall, ICG-A provides better predictive accuracy than FA and clinical judgment [5].

More recently, other methods based on new technologies have been used to evaluate perfusion both clinically and experimentally. These include scDCT, a near-infrared spectroscopy device used to measure tissue oxygen saturation and hemoglobin concentration, and thermal imaging with a FLIR device [12,15,47,61,62]. HSI is an alternative to ICG-A; it can accurately assess perfusion and allows for safer and more predictable results to be obtained in breast reconstruction [13,14,24,30,65]. Other methods include a clinical assessment tool with eight binary questions and intraoperative thickness measuring (ultrasound) of the flap to predict ischemic complications [45,46].

One of the limitations of this review was the significant heterogeneity among the included studies, which made conducting a meta-analysis unfeasible. Also, not all the PRISMA criteria could be reached, although many of them were. The study populations were inconsistent, mastectomy skin flap perfusion assessment techniques varied widely, and the experimental models differed. While this highlights the versatility of mastectomy skin flap perfusion assessment techniques, it also complicates the process of drawing objective conclusions. Additionally, this review was limited by its focus on studies from the past 10 years and those published in English. Gray literature was not actively sought. However, we believe that these methodological constraints should not affect the overall conclusions of the analysis. Finding a suitable perfusion assessment method is still a subject of interest, and the abundance of technological advances can only expand the field for future research. Clinical evaluation (Method 1) is a mandatory tool and should not be underestimated in any event. Perfusion assessment tools complement and should not replace clinical evaluation.

## 5. Conclusions

To conclude, among all the four methods (clinical examination and instrumental methods) used to evaluate skin perfusion after mastectomy, ICG-A seems to be the preferred and the most widely used; it has more advantages, especially in high-risk patients, while new technologies show promising results, with notable ease of use, and might be of great interest in the future. Finally, it is important to understand that although other methods based on less-invasive techniques (spectroscopy and thermal imaging) have proven their utility, they should only complement and not replace clinical evaluation.

## Figures and Tables

**Table 1:** Summary of the perfusion assessment methods.

	Method	Description
1	Clinical examination	Observation of skin color, capillary refill time, bleeding
2	Angiography (ICG and fluorescein)	Intravenously injected dye (ICG or fluorescein)
3	Spectroscopy (near infrared oximetry, HSI)	Measures tissue oxygenation, water content, hemoglobin concentration
4	Thermal imaging	Measures skin surface temperature

**Table 2:** Summary of review papers.

Name	Method of Perfusion Assessment	Description
Griffiths et al. (2016) [19]	Method 2	Useful in high-risk patients (smokers, high BMI, large breasts)
Sbitany et al. (2017) [23]	Method 1, Method 2	Clinical examination coupled with ICG-A can enhance the accuracy
Jeon et al. (2018) [5]	Method 1, Method 2	ICG-A provides better predictive accuracy than FA and clinical judgment
Srinivasa et al. (2019 [21]	Method 1, Method 2	ICG-A can be used if concerns arise with clinical examination
Khavanin et al. (2019) [8]	Method 2, Method 3	Pros and cons of each method is described
Liu et al. (2019) [22]	Method 1, Method 2	ICG-A is more reliable and more objective than clinical examination, with a lower complication rate
Johnson et al. (2020) [25]	Method 1, Method 2	ICG-A is superior to clinical judgment but cannot replace it
Driessen et al. (2020) [26]	Method 2	ICG-A aids in the surgeons’ decision-making
Fleischl et al. (2020) [27]	Method 2	Reduction of flap necrosis rates after the implementation of ICG-A
da Silva Neto et al. (2020) [28]	Method 2	ICG-A allows the surgeon to assess perfusion and achieve the best outcomes
Pruimboom et al. (2020) [14]	Method 2	ICG-A is a helpful tool, although randomized controlled studies are needed
Pruimboom et al. (2020) [29]	Method 2	Describes factors that influence and optimize ICG-A intensity
Velazquez et al. (2020) [18]	Method 2	Describes the utility of FA
Chopra et al. (2021) [20]	Method 1, Method 2	Clinical examination coupled with ICG-A can enhance the accuracy
Hren et al. (2022) [13]	Method 3	Describes the use of HSI in mastectomy skin flap perfusion
Pruimboom et al. (2022) [14]	Method 3	Describes the use of HSI in mastectomy skin flap perfusion
Schols et al. (2022) [30]	Method 2	ICG-A is the most frequently used method and should no longer be considered experimental
Meshkin et al. (2023) [17]	Method 2	Important key decision-making tools in reducing necrosis
Doremus et al. (2024) [31]	Method 1, Method 2	Clinical assessment is critical for flap perfusion, and the use of adjuncts, such as ICG-A, has become a staple of breast reconstruction

**Table 3:** Summary of clinical studies.

Name	Method of Perfusion Assessment	Description
Duggal et al. (2014) [11]	Method 2	Decreased incidence of MSFN and unexpected reoperations; cost effective
Munabi et al. (2014) [48]	Method 2	ICG-A predicts postoperative outcomes with high accuracy and serves as a useful adjunct to other intra-operative parameters to evaluate perfusion
Rinker et al. (2016) [49]	Method 1, Method 2	Comparison between direct visualization to FA and ICG-A; FA was associated with the lowest rate of complications, but ICG-A was also shown to reduce mastectomy flap necrosis compared with direct visualization
Diep et al. (2016) [32]	Method 1, Method 2	Lower rate of sever flap necrosis with ICG-A compared to clinical judgment
Harless et al. (2016) [26]	Method 2	Reduction in complication rate from 13.8% to 6.6% after implementation of ICG-A
Mattison et al. (2016) [41]	Method 1, Method 2	Compared to clinical examination, ICG-A would result in a more aggressive resection and does not play a role in low-risk cases; it should be used when the clinical assessment is marginal as it tends to overpredict necrosis
Jones et al. (2017) [50]	Method 2	Single-stage DTI breast reconstruction, as an alternative to a staged approach, is safer with ICG-A
Gorai et al. (2017) [40]	Method 1, Method 2	Compared to clinical judgment, ICG-A-guided skin-trimming reduces the rate of deep-skin necrosis requiring additional treatment
Venturi et al. (2017) [36]	Method 1, Method 2	ICG-A is useful in identifying patients at risk for nipple necrosis when perfusion is difficult to assess clinically
Geletzke et al. (2018) [37]	Method 2	ICG-A was performed before the mastectomy to assess the dominant vasculature of the skin flap and preserve the mapped vessels; this is correlated with reduced complications, especially in high-risk patients, and improved patient outcomes
Mirhaidari et al. (2018) [51]	Method 2	MSFN rates were lower with ICG-A compared to previously published data
Wang et al. (2018) [50]	Method 2	ICG-A was used to investigate nipple–areola complex perfusion and the appropriate incision placement for nipple sparing mastectomy
Yang et al. (2018) [52]	Method 2	ICG-A was used to analyze the changes in tissue perfusion in conjunction with the tension level during implant-based reconstruction
Frey et al. (2019) [45]	Method 1	Description of a clinical risk assessment tool consisting of eith binary questions, useful when ICG-A is not available; higher scores are associated with greater risk of ischemic complications
Diep et al. (2019 [33]	Method 2	Improved outcomes after ICG-A: fewer ischemic complications, increased expander fill volumes, shorter waiting time for final reconstruction
Kim et al. (2019) [53]	Method 2	Description of a quantitative assessment of nipple perfusion rate with ICG-A to guide the decision-making process intraoperatively
Balci et al. (2019) [54]	Method 1, Method 2	ICG-A is a better predictor for skin necrosis than conventional assessment and is used to predict and locate poorly perfused areas in patients undergoing reconstruction following nipple-sparing mastectomy
Damsgaard et al. (2019) [55]	Method 2	A scoring system to evaluate perfusion using ICG-A; tissue with a value < 33% is excised to prevent ischemic complications and revision surgery
Parus et al. (2020) [38]	Method 2	ICG-A was used to evaluate the perfusion of a infra mammary fold flap for a safe reconstruction and avoidance of complications
Koonce et al. (2020) [43]	Method 2	Description of four types of ischemia patterns on ICG-A and algorithm for management
Pestana et al. (2021) [44]	Method 2	FA is used to assess mastectomy skin perfusion and likely limits mastectomy complication effects on reconstruction
Luze et al. (2022) [15]	Method 4	Thermal imaging with a FLIR device can be useful in evaluating perfusion; skin areas with a temperature lower than 26 degrees Celsius are highly likely to develop subsequent ischemic complications
Nguyen et al. (2022) [34]	Method 2	ICG-A is useful as a supplement rather than a substitute for clinical assessment
Nguyen et al. (2022) [35]	Method 2	ICG-A may be most useful on high-risk patients (smokers, high BMI, large breasts)
Nguyen et al. (2022) [56]	Method 2	Distinct patterns of ischemia and low perfusion values with ICG-A may be used in the decision to delay reconstruction
Pruimboom et al. (2022) [14]	Method 3	HSI could potentially accurately assess the mastectomy skin flap perfusion, although the study was conducted on a small sample size
Mazdeyasna et al. (2022) [47]	Method 3	Speckle contrast diffuse correlation tomography (scDCT) is a noninvasive and inexpensive alternative to ICG-A for the intraoperative assessment of perfusion in mastectomy flaps
Moritz et al. (2023) [12]	Method 3	SnapshotNIR device utilizes near-infrared spectroscopy to measure tissue oxygen saturation and hemoglobin concentration and can be used to evaluate tissue perfusion after mastectomy
Nguyen et al. (2023) [39]	Method 2	Increased tendency to delay the reconstruction after implementation of ICG-A; more information about perfusion led to a more conservative approach
Lauritzen et al. (2023) [42]	Method 2	In oncoplastic breast surgery, ICG-A was used to assess the perfusion intraoperatively, as well as for the identification and selection of perforators, and this led to no patients developing skin necrosis
Pagliara et al. (2023) [46]	Method 1	Flap thickness ratio—intraoperative thickness (ultrasound) compared to preoperative data (digital mammograms)—indicated a lower value in ischemic complications
Jindal et al. (2023) [56]	Method 2	Recent advances have explored the role of artificial intelligence in developing surgical recommender systems; machine learning algorithms were applied on ICG-A videos evaluating mastectomy flap perfusion and achieved an acceptable standard, encouraging further optimization and validation for intraoperative decision-support tools

## Data Availability

Not applicable.
